# Transcriptional response to Wnt activation regulates the regenerative capacity of the mammalian cochlea

**DOI:** 10.1242/dev.166579

**Published:** 2018-11-27

**Authors:** Anshula Samarajeewa, Danielle R. Lenz, Lihong Xie, Hao Chiang, Rory Kirchner, Joanna F. Mulvaney, Albert S. B. Edge, Alain Dabdoub

**Affiliations:** 1 Department of Laboratory Medicine and Pathobiology, University of Toronto, Toronto ON, M5S 1A8, Canada; 2 Department of Otolaryngology, Harvard Medical School, Boston, MA 02114, USA; 3 Eaton-Peabody Laboratory, Massachusetts Eye and Ear, Boston, MA 02114, USA; 4 Biological Sciences, Sunnybrook Research Institute, Sunnybrook Health Sciences Centre, Toronto ON, M4N 3M5, Canada; 5 Department of Otolaryngology-Head and Neck Surgery, First Affiliated Hospital of Guangxi Medical University, Nanning Guangxi, 530021, China; 6 Department of Biostatistics, Harvard T.H. Chan School of Public Health, Boston, MA 02115, USA; 7 Department of Otolaryngology - Head & Neck Surgery, University of Toronto, Toronto ON, M5G 2C4, Canada

**Keywords:** Mouse, Wnt, Notch, Hearing, Regeneration, Plasticity

## Abstract

Lack of sensory hair cell (HC) regeneration in mammalian adults is a major contributor to hearing loss. In contrast, the neonatal mouse cochlea retains a transient capacity for regeneration, and forced Wnt activation in neonatal stages promotes supporting cell (SC) proliferation and induction of ectopic HCs. We currently know little about the temporal pattern and underlying mechanism of this age-dependent regenerative response. Using an *in vitro* model, we show that Wnt activation promotes SC proliferation following birth, but prior to postnatal day (P) 5. This age-dependent decline in proliferation occurs despite evidence that the Wnt pathway is postnatally active and can be further enhanced by Wnt stimulators. Using an *in vivo* mouse model and RNA sequencing, we show that proliferation in the early neonatal cochlea is correlated with a unique transcriptional response that diminishes with age. Furthermore, we find that augmenting Wnt signaling through the neonatal stages extends the window for HC induction in response to Notch signaling inhibition. Our results suggest that the downstream transcriptional response to Wnt activation, in part, underlies the regenerative capacity of the mammalian cochlea.

## Abstract (precis)

**Summary:** Canonical Wnt activation in the mammalian cochlea elicits a unique, age-dependent transcriptional response, which in part regulates the regenerative capacity of supporting cells during cochlear maturation.

## INTRODUCTION

Noise-induced and age-related degeneration of sensory hair cells (HCs) is a leading cause of hearing deficits. Owing to a lack of regenerative capacity in the adult mammalian cochlea, damage to HCs is irreversible. In contrast to adults, neonatal mice have a limited capacity to regenerate HCs (Bramhall et al., 2014; Chai et al., 2012; Shi et al., 2013, 2012; Yamamoto et al., 2006). Consequently, there is considerable interest in defining the temporal pattern of this regenerative response and its underlying mechanisms in neonates.

By birth, cellular organization of the cochlea is complete, with the sensory epithelium organized into one row of inner HCs, three rows of outer HCs and interdigitating non-sensory supporting cells (SCs). In response to modulation of specific signaling pathways, SCs in the neonatal cochlea function as endogenous stem cells and HC regeneration proceeds through either direct transdifferentiation or proliferation of SCs and subsequent transdifferentiation to HCs (Cox et al., 2014). Direct transdifferentiation comes at the cost of SC loss; thus, replenishing SCs is crucial for auditory function.

Two key pathways that can be modulated to stimulate these endogenous stem cells are canonical Wnt and Notch pathways (Żak et al., 2015). Canonical Wnt signaling is activated when secreted Wnts bind Frizzled receptors, causing a downregulation of GSK3β activity and subsequent stabilization of cytoplasmic β-catenin. Within the nucleus, stabilized β-catenin binds TCF/Lef transcription factors to activate target genes (Clevers, 2006). During embryonic stages, Wnt signaling plays an essential role in both prosensory cell proliferation and HC differentiation (Jacques et al., 2012; Shi et al., 2014). Similarly, Notch signaling is important for cell fate determination and generates the mosaic of interdigitating HCs and SCs (Kiernan, 2013). Notch signaling is initiated when membrane-localized Delta and Jagged ligands bind Notch receptors. Ligand-receptor interaction results in enzymatic cleavage of the Notch receptor by γ-secretase and subsequent release of the Notch receptor intracellular domain that activates downstream target genes.

Inhibition of Notch signaling following birth induces transdifferentiation of SCs to HCs; however, this ability to transdifferentiate is lost by postnatal day (P) 3 (Maass et al., 2015). Similarly, forced Wnt activation following birth both *in vitro* and *in vivo* can promote SC proliferation and induction of new HCs (Chai et al., 2012; Kuo et al., 2015; Shi et al., 2013, 2012). Interestingly, SCs also lose their ability to respond to Wnt with age and Wnt activation fails to produce the same regenerative effect in adults (Shi et al., 2013). Understanding this response to Wnt during early developmental stages has the potential to guide strategies for HC regeneration in adults. In this study, we have investigated the temporal pattern and underlying mechanisms of the regenerative response to Wnt activation at different stages of development in the mammalian cochlea.

## RESULTS

### Wnt activation promotes proliferation of SOX2-positive cells at embryonic and early neonatal stages

We first sought to evaluate the temporal pattern of the regenerative response to canonical Wnt activation in the post-mitotic cochlea in terms of proliferation and HC induction. During mouse cochlear development, SOX2^+^ prosensory cells exit the cell cycle at embryonic day (E) 12.5 and subsequently differentiate into HCs or SOX2^+^ SCs (Chen and Segil, 1999). We therefore established cochlear explant cultures following terminal mitosis at E13.5, P0 and P5. We activated Wnt for 5 days *in vitro* (DIV) using CHIR99021 (CHIR), a selective inhibitor of GSK3β function, and supplemented the media with the proliferation marker BrdU for the entire culture period. Control explants were cultured with DMSO and BrdU. After fixation, explants were immunolabeled for SOX2 (early prosensory or late SC marker), BrdU (proliferation marker) and myosin VI (MYOVI) or myosin VIIA (MYOVIIA; HC markers). Co-labelling with SOX2 and BrdU was used to quantify proliferation.

**Fig. 1.**
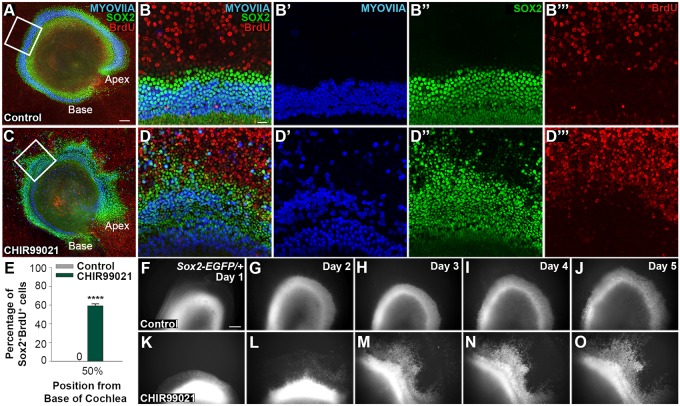
**Wnt activation at E13.5 induces proliferation and expansion of SOX2-positive cells.** (A) Low- and (B-B‴) high-magnification views of E13.5 cochlear explants cultured with control media and BrdU for 5 DIV shows immunostaining for MYOVIIA^+^ HCs (blue), SOX2^+^ prosensory cells (green) and proliferation marker BrdU (red). HCs developed normally and SOX2^+^BrdU^+^ cells were absent in controls. (C) Low- and (D-D‴) high-magnification views of E13.5 explants cultured with CHIR and BrdU for 5 DIV show an increase in SOX2^+^BrdU^+^ cells and the number of differentiated HCs. Boxed regions in A and C are magnified in B-B‴ and D-D‴, respectively. (E) Quantification of SOX2^+^BrdU^+^ cells within a 200×200 μm box positioned over the HC domain at the 50% position from the base revealed a statistically significant increase in SOX2^+^BrdU^+^ cells in E13.5 explants cultured with CHIR compared with controls. *n*=3 independent litters; two-tailed Student's *t*-test, ****P<0.0001. Live-imaging of *Sox2^EGFP/+^* cochlear explants. (F-J) Organotypic E13.5 cochlear explant cultured with control media over 5 days. Sequential images show growth at mid-base at every 24 h, starting at E14.5 (day 1). (K-O) E13.5 explant cultured with CHIR over 5 days shows a lateral expansion of the SOX2^+^ domain. Scale bars: 100 µm in A,C,F-O; 20 µm in B-B‴,D-D‴.

At E13.5, SOX2^+^ prosensory cells are typically quiescent (Chen and Segil, 1999; Lee et al., 2006). Control explants established at E13.5 lacked SOX2^+^BrdU^+^ cells after 5 DIV, indicating that SOX2^+^ cells remained mitotically quiescent (Fig. 1A,B,E). However in agreement with reports using other Wnt agonists (Jacques et al., 2012), Wnt activation with CHIR at E13.5 resulted in proliferation of SOX2^+^ prosensory cells, as demonstrated by a statistically significant increase in SOX2^+^BrdU^+^ cells (Fig. 1C-E). Wnt activation at E13.5 also resulted in a statistically significant increase in the number of differentiated HCs (166±9.45) compared with controls (104±4.75), as assessed by the number of MYOVIIA^+^ cells at the 50% position from the base of the cochlea (*n*=3 independent litters, two-tailed Student's *t*-test, *P*<0.01). Live imaging of an E13.5 cochlear explant from a *Sox2^EGFP/+^* reporter mouse further illustrates the effects of Wnt activation on prosensory cells. In contrast to controls (Fig. 1F-J), SOX2^+^ cells expanded into the lateral region following Wnt activation (Fig. 1K-O) over a 5-day culture period.

Additionally, we immunostained E13.5 cultured explants for the Notch-ligand jagged 1 (JAG1), another early prosensory and late SC marker. Wnt activation at E13.5 resulted in an expanded JAG1^+^ domain throughout the cochlea, as well as diminished membrane localization of E-cadherin and increased expression of cyclin D1 in SOX2^+^ cells (Fig. S1). Furthermore, when we established cochlear explants at E13.5 and activated Wnt on E15.5 for 4 days, an expanded JAG1^+^ domain was observed at the apex (Fig. S2).

**Fig. 2.**
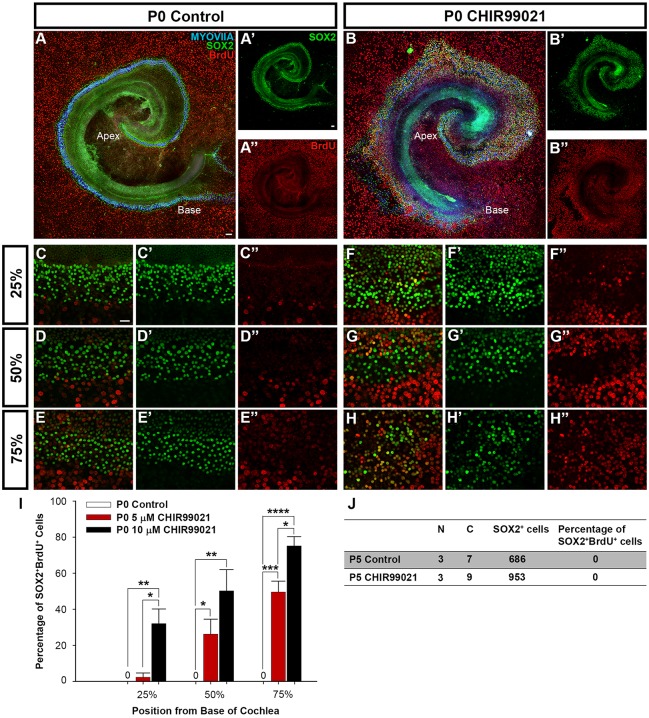
**Wnt activation promotes proliferation of SOX2-positive cells at early neonatal stages.** (A-B″) Low-magnification view of P0 explants cultured with (A-A″) control media or (B-B″) CHIR and BrdU for 5 DIV. Explants were immunostained for MYOVI^+^ HCs (blue), SOX2^+^ SCs (green) and BrdU (red). Treatment with CHIR resulted in a lateral expansion of the sensory epithelium. (C-E″) High-magnification views of (C-C″) base, (D-D″) mid-base and (E-E″) apex of P0 explant cultured with control media and BrdU for 5 DIV. SOX2^+^BrdU^+^ cells were absent in controls. (F-H″) High-magnification view of (F-F″) base, (G-G″) mid-base and (H-H″) apex of P0 explant cultured for 5 days with 10 µM CHIR and BrdU shows an increase in SOX2^+^BrdU^+^ cells. (I) Quantification of SOX2^+^BrdU^+^ cells within 200 µm segments along the length of the cochlea and entire width of the sensory epithelium at positions 25%, 50% and 75% from the base in P0 explants cultured with 5 µM CHIR revealed a statistically significant increase in SOX2^+^BrdU^+^ cells compared with controls. Treatment with 10 µM CHIR resulted in a more robust and statistically significant increase SOX2^+^BrdU^+^ cells compared with controls and treatment with 5 µM CHIR. *n*=4 independent litters; two-tailed Student's *t*-test, *P<0.05, **P<0.01, ***P<0.001, ****P<0.0001. (J) Quantification of SOX2^+^BrdU^+^ cells within a 200 µm segment along the length of the cochlea and entire width of the sensory epithelium at the 75% position from the base in P5 explants cultured with control media or 10 µM CHIR for 5 DIV showed a lack of SOX2^+^BrdU^+^ cells under both conditions. *n*=3 independent litters and a total of seven or nine cochleae counted in control or CHIR-treated samples, respectively; two-tailed Student's *t*-test, *P*=1. Scale bars: 100 µm in A-B″; 20 µm in C-H″.

In the neonatal cochlea, Wnt activation promoted the proliferation of SOX2^+^ SCs at P0; however, this proliferative response was no longer observed by P5. By P0, HC and SC differentiation is complete but the cochlea continues to mature until the onset of hearing at P14. In control explants established at P0, BrdU incorporation by SOX2^+^ cells was not observed, indicating that SOX2^+^ cells remained mitotically quiescent (Fig. 2A,C-E,I). However, Wnt activation at P0 with 5 μM CHIR resulted in a statistically significant increase in SOX2^+^BrdU^+^ cells compared with controls in middle and apical segments of the cochlea after 5 DIV (Fig. 2I). Treatment with a higher dose of 10 µM CHIR resulted in a more robust and statistically significant increase in SOX2^+^BrdU^+^ cells throughout the cochlea from base to apex compared with controls and compared with treatment with 5 μM CHIR (Fig. 2B,F-I). At both concentrations, an increasing gradient of SOX2^+^BrdU^+^ cells was observed from base to apex (Fig. 2I).

As 10 μM CHIR induced a more robust proliferative response at P0, we used this dose to activate Wnt at P5. However, Wnt activation at P5 with 10 μM CHIR for 5 DIV failed to induce proliferation in SOX2^+^ cells (lack of BrdU incorporation by SOX2^+^ cells; Fig. 2J). Modulating the concentration of CHIR from 1.5 μM to 20 μM also failed to induce proliferation in SOX2^+^ cells at P5 (data not shown).

### Stimulation of Wnt signaling promotes similar levels of β-catenin accumulation in both embryonic and postnatal cochleae

As Wnt activation promoted SC proliferation at P0 but not P5, we hypothesized that this age-dependent decline in proliferation could result from decreased β-catenin accumulation, diminished TCF/Lef complex activation and/or changes in the transcriptional response to Wnt by P5. First, in order to determine whether there is decreased β-catenin accumulation at P5 compared with earlier developmental stages, we quantified β-catenin in embryonic and postnatal cochleae following transient exposure to the Wnt activator. We treated E13.5, P0 and P5 cochleae with DMSO or 10 μM CHIR for 3 h and subjected whole-cochlear lysates to western blotting. Membranes were immunoblotted with active β-catenin, β-catenin and GAPDH (loading control) antibodies. Active β-catenin antibody selectively recognizes the stabilized form of β-catenin, which is dephosphorylated at Ser37 and/or Thr41.

**Fig. 3.**
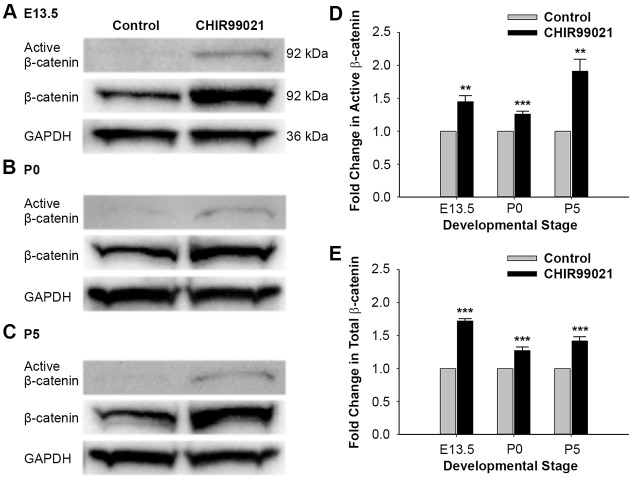
**Wnt activation promotes stabilization and accumulation of active β-catenin and total β-catenin in E13.5, P0 and P5 cochleae.** (A-C) Immunoblots showing accumulation of active β-catenin and total β-catenin in (A) E13.5, (B) P0 and (C) P5 cochleae following exposure to control media or CHIR for 3 h. (D,E) Quantification of (D) active β-catenin and (E) total β-catenin in immunoblots (mean±s.e.m.) revealed a statistically significant increase in both active and total β-catenin accumulation at all three developmental stages following Wnt activation. *n*=3 independent litters at E13.5, P0 and P5; two-tailed Student's *t*-test, **P<0.01, ***P<0.001.

Compared with controls, transient exposure of E13.5 cochlear explants to CHIR resulted in increased accumulation of both active β-catenin and total β-catenin after 3 h (Fig. 3A). This pattern of active β-catenin and total β-catenin accumulation in response to CHIR did not appear to be different at either P0 or P5 (Fig. 3B,C). Furthermore, densitometric analysis of immunoblots revealed a robust and statistically significant increase in both active and total β-catenin accumulation following exposure to the Wnt activator at all three developmental stages (Fig. 3D,E).

### Stimulation of Wnt signaling enhances TCF/Lef complex activation at both early and late neonatal stages

As exposure to the Wnt activator promoted β-catenin accumulation at all three developmental stages, we aimed to evaluate whether the age-dependent decline in proliferation could be attributed to diminished TCF/Lef complex activation in the P5 cochlea. To detect TCF/Lef complex activation, we cultured P1 and P5 cochlear explants from *TCF/Lef:H2B-GFP* reporter mice (Ferrer-Vaquer et al., 2010) with DMSO or 3 μM CHIR for 13 h and analyzed GFP expression. A 13 h time-point was chosen to prevent potential upregulation of GFP signal as a result of culture conditions instead of CHIR treatment. Subpopulations of SCs, including inner border, inner phalangeal, pillar, Deiters', Hensen's and Claudius cells, were identified based on SOX2 labeling and localization along the mediolateral axis of the sensory epithelium. HCs were identified based on MYOVIIA labeling. Co-labeling with GFP and SOX2 or GFP and MYOVIIA was used to detect TCF/Lef complex activation in SCs or HCs, respectively.

**Fig. 4.**
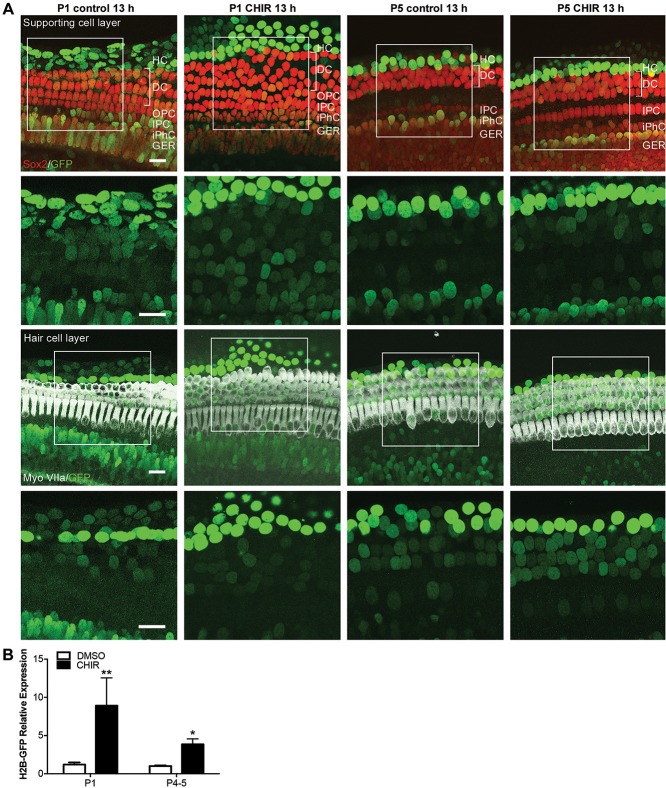
**Activation of the TCF/Lef complex in the sensory epithelium is enhanced following exposure to the Wnt activator at both P0 and P5.** (A) P1 and P5 cochleae from Wnt-reporter mice cultured with control media or CHIR for 13 h *in vitro* show a GFP signal and immunostaining for MYOVIIA^+^ HCs (white) and SOX2^+^ SCs (red)*.* Control samples at P1 and P5 showed native Wnt activation in SCs and HCs, as demonstrated by a positive GFP signal. Exposure to CHIR increased this GFP signal further at both P1 and P5. Boxed region in merged images indicates where the enlarged GFP-only image is taken from. Scale bars: 20 μm. (B) Expression analysis of H2B-GFP using qPCR demonstrates a significant upregulation of H2B-GFP in explants after CHIR treatment at both P1 and P5 (*P*<0.05). Results are presented as average fold change compared with control ±s.e.m. *n*=3 independent litters at P0 and P5; two-tailed Student's *t*-test, *P<0.05, **P<0.01.

Control samples showed native activation of the Wnt pathway in HCs and a subset of SCs at both P1 and P5 (Fig. 4A). GFP expression at P1 was strongest in the Hensen's and Claudius cells but could also be detected in inner border, inner phalangeal, and both inner and outer HCs. This expression pattern did not appear to differ between P1 and P5. Compared with controls, exposure of both P1 and P5 cochlear explants to CHIR increased the GFP signal further and induced low-level GFP expression in Deiters' cells after 13 h. Analysis of the H2B-GFP reporter using qPCR supported this finding, demonstrating significant increase of H2B-GFP at both P1 and P5 after CHIR treatment, with no significant difference between treated samples at P1 compared with P5 (Fig. 4B). These results indicate that the Wnt pathway is endogenously active and that exogenous Wnt stimulation further enhances TCF/Lef complex activation in a similar manner at both P1 and P5, though this activation is insufficient to induce SC proliferation in the P5 cochlea.

### Newly proliferated SCs in the neonatal cochlea are competent to transdifferentiate to HCs up to at least P8

Recent studies have demonstrated that Notch signaling inhibition with the γ-secretase inhibitor DAPT promotes the transdifferentiation of SCs to HCs in the neonatal cochlea but only up to P3 (Maass et al., 2015; Yamamoto et al., 2006). Here, we show that newly proliferated SCs in the neonatal cochlea can transdifferentiate to HCs in response to Notch inhibition up to at least P8, thus extending the window for HC induction.

We first cultured P0 cochlear explants with CHIR and BrdU for 5 DIV, prior to treatment with DAPT for 3 DIV. Control explants were cultured with DMSO and BrdU before exposure to DAPT. Explants were fixed and immunolabeled for BrdU, SOX2 and MYOVI. Co-labeling with MYOVI and BrdU was used to quantify newly generated HCs.

**Fig. 5.**
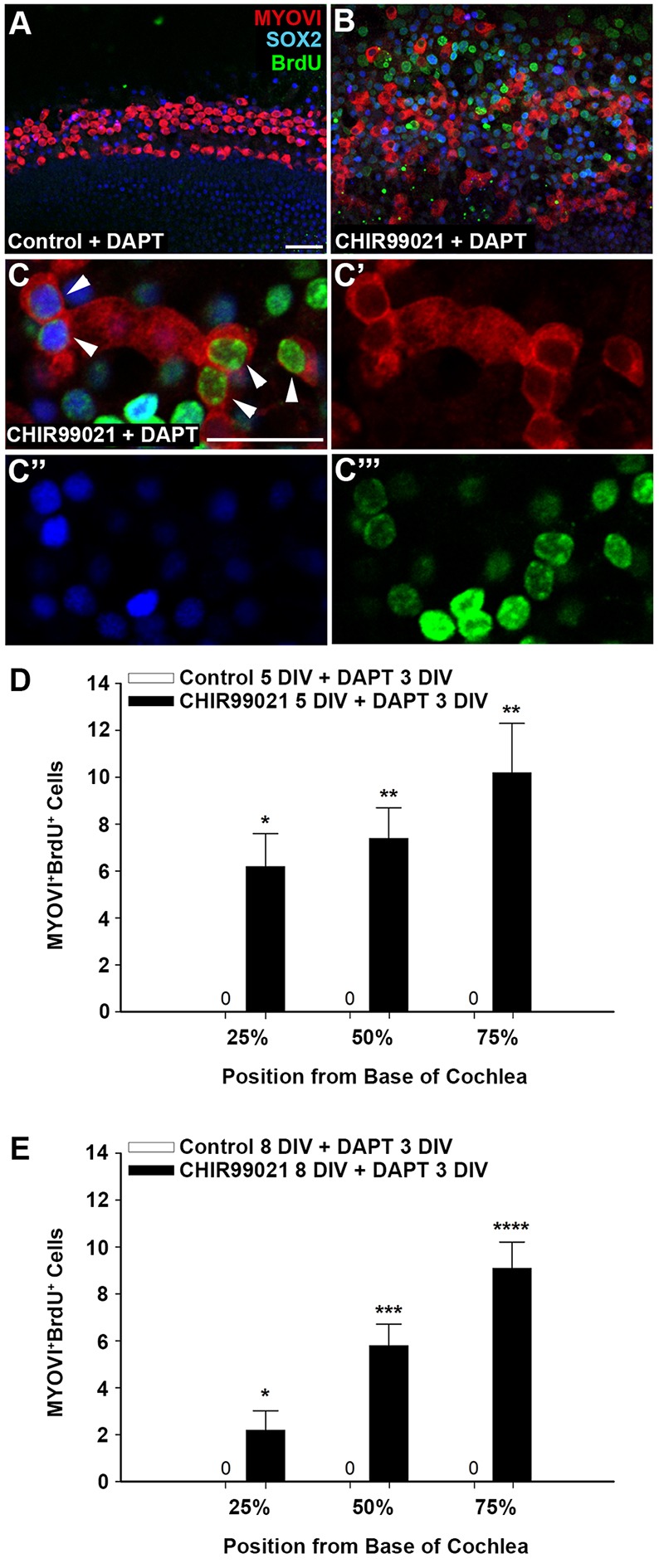
**Wnt-induced SOX2-positive cells are competent to transdifferentiate to HCs in response to Notch inhibition up to at least P8.** (A,B) Low-magnification views (at 50% from base) of P0 explants cultured with (A) control media or (B) CHIR and BrdU for 5 days, followed by the Notch inhibitor DAPT for 3 days show immunostaining for MYOVI^+^ HCs (red), SOX2^+^ cells (blue) and BrdU (green). (C-C‴) High-magnification view of P0 explant cultured with CHIR and BrdU for 5 days, followed by DAPT for 3 days. MYOVI^+^BrdU^+^ cells were absent in controls. Wnt activation at P0 and subsequent Notch inhibition on P5 generated new MYOVI^+^BrdU^+^ HCs (arrowheads). (D,E) Wnt activation with CHIR for (D) 5 DIV and (E) 8 DIV, followed by Notch inhibition with DAPT for 3 DIV resulted in a statistically significant increase in newly generated MYOVI^+^BrdU^+^ HCs compared with controls, which lacked MYOVI^+^BrdU^+^ cells. Quantification of MYOVI^+^BrdU^+^ cells was conducted within 200 µm segments along the length of the cochlea and the entire width of the sensory epithelium at positions 25%, 50% and 75% from the base. *n*=4 independent litters; two-tailed Student's *t*-test, *P<0.05, **P<0.01, ***P<0.001, ****P<0.0001. Scale bars: 20 µm in A-C‴.

Wnt activation at P0, followed by Notch inhibition at a stage equivalent to P5 resulted in proliferation of SOX2^+^ cells and subsequent transdifferentiation of a subset of these into HCs, generating a statistically significant number of MYOVI^+^BrdU^+^ cells (Fig. 5B-D). We found that we could further extend this window for HC induction up to P8, as P0 cochlear explants cultured with CHIR and BrdU for 8 DIV, prior to treatment with DAPT for three additional days also generated a statistically significant number of MYOVI^+^BrdU^+^ cells (Fig. 5E). An increasing gradient of MYOVI^+^BrdU^+^ cells was observed from base to apex (Fig. 5D,E). SOX2^+^BrdU^+^ cells were also observed in CHIR- and DAPT-treated explants, indicating that not all Wnt-induced SOX2^+^ cells transdifferentiated to HCs (Fig. 5B,C). In contrast, MYOVI^+^BrdU^+^ cells or SOX2^+^BrdU^+^ cells were not observed in control explants cultured with DMSO prior to treatment with DAPT, indicating that SOX2^+^ cells failed to proliferate and subsequently transdifferentiate to HCs (Fig. 5A,D,E). Our results suggest that augmenting Wnt activation in the neonatal stages not only replenishes the SC population but also extends the window for HC induction up to at least P8, thus generating new HCs in the maturing cochlea.

### Augmenting Wnt activation through the neonatal stages alters expression of specific Wnt and Notch pathway genes

We hypothesized that Wnt activation at P0 confers competency for SOX2^+^ cell proliferation and subsequent transdifferentiation to HCs by altering the expression of specific Wnt and Notch pathway genes. To evaluate this, we cultured P0 cochlear explants with DMSO or CHIR and extracted total RNA from whole-cochlear lysates after 5 DIV. We assessed the relative expression of Wnt and Notch pathway genes between control and experimental samples using Qiagen RT^2^-profiler PCR Arrays.

Compared with controls, exposure to CHIR resulted in statistically significant changes in the expression of 18 Wnt pathway genes in the P0 cochlea after 5 DIV (Table 1). Interestingly, the transcription factor gene *Tcf1*, which is thought to promote self-renewal, was upregulated and *Tcf3,* which has an inhibitory effect on proliferation, was downregulated (Abbas and Dutta, 2009). Expression of the biomarker of canonical Wnt signaling gene *Axin2* as well as the Wnt-modulator and SC marker gene *Kremen1* (Mulvaney et al., 2016) was upregulated.

**Table 1.**
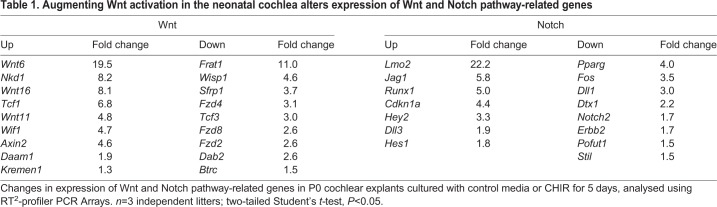
**Augmenting Wnt activation in the neonatal cochlea alters expression of Wnt and Notch pathway-related genes**

Exposure to CHIR also resulted in statistically significant changes in the expression of 15 Notch-pathway genes (Table 1). The SC markers *Jag1*, *Hes1* and *Hey2* were upregulated, and *Notch2* was significantly downregulated. Furthermore, with respect to cell-cycle genes, the cyclin-dependent kinase inhibitor *Cdkn1a* (Xiong et al., 1993) was upregulated and the cell-cycle regulator *Stil* (Izraeli et al., 1997) was downregulated.

**Fig. 6.**
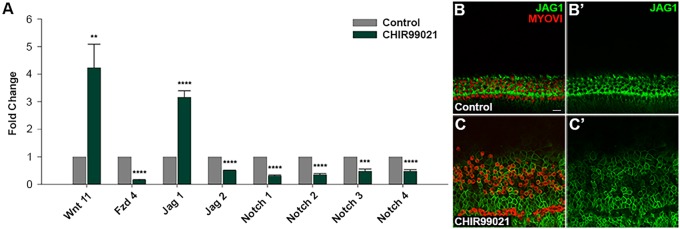
**Validation of relative mRNA expression of select Wnt and Notch pathway genes using RT-qPCR.** (A) RT-qPCR analysis of relative mRNA expression of select Wnt and Notch pathway genes in P0 explants cultured with control media or CHIR for 5 days. *n*=3 independent litters; two-tailed Student's *t*-test, **P<0.01, ***P<0.001, ****P<0.0001. (B-C′) High-magnification view of P0 explant cultured with (B,B′) control media or (C-C′) Wnt activator for 5 days shows immunostaining for MYOVI^+^ HCs (red), JAG1^+^ SCs (green). Wnt activation at P0 resulted in a lateral expansion of the JAG1^+^ domain. Scale bar: 20 µm.

We used real-time quantitative PCR (RT-qPCR) to validate changes in expression of Notch ligands *Jag1* and *Jag2* and Notch receptors *Notch1*, *Notch2*, *Notch3* and *Notch4* that were observed with RT^2^-profiler PCR Arrays. Expression of *Jag1* was upregulated following Wnt activation (Fig. 6A). In agreement with this finding, immunolabeling of P0 explants demonstrated a laterally expanded JAG1^+^ domain when cultured with CHIR for 5 days (Fig. 6B,C). Expression of *Jag2*, which is found in nascent HCs of the developing cochlea, and that of the Notch receptors *Notch1*, *Notch2*, *Notch3* and *Notch4* was downregulated in response to Wnt activation (Fig. 6A). In the initial RT^2^-profiler PCR Arrays, *Jag2* expression was unchanged and, although *Notch1*, *Notch2*, *Notch3* and *Notch4* were downregulated following Wnt activation, only the decrease in *Notch2* expression was statistically significant. In addition, we used RT-qPCR to validate changes in expression of the Wnt pathway genes *Wnt11* and *Fzd4* that were observed with RT^2^-profiler PCR Arrays. In agreement with results from RT^2^-profiler PCR Arrays, RT-qPCR showed an increase in expression of *Wnt11* and a decrease in expression of *Fzd4* following Wnt activation (Fig. 6A).

These results demonstrate that maintaining high levels of Wnt activation in the neonatal stages alters the expression of specific ligands, receptors, modulators and downstream target genes of Wnt and Notch signaling pathways. This may play a role in SC proliferation and confer competency for HC induction in response to Notch inhibition.

### The gene expression profile of the sensory epithelium following β-catenin stabilization *in vivo* differs between P1 and P4

Our *in vitro* experiments demonstrated that Wnt activation using CHIR promotes SC proliferation at P0, but fails to produce the same effect at P5. As β-catenin accumulation and Wnt reporter activity appear to have a similar pattern at both P0 and P5, we sought to compare the broad transcriptional response to Wnt activation between early and late neonatal time-points using RNA sequencing. To exclude off-target effects of the GSK3β inhibitor, we chose to activate Wnt using our genetic mouse model *Sox2-CreER; β-catenin^flox(Exon3)/+^*. Exon 3 of *Ctnnb1* contains the GSK3β phosphorylation site, which marks β-catenin for degradation. When Cre is activated with tamoxifen, exon 3 is excised and β-catenin is stabilized specifically in SOX2^+^ cells. We have previously shown that Wnt activation using genetic β-catenin stabilization promotes the proliferation of SOX2^+^ cells at P1 but not in the adult cochlea (Shi et al., 2013).

**Fig. 7.**
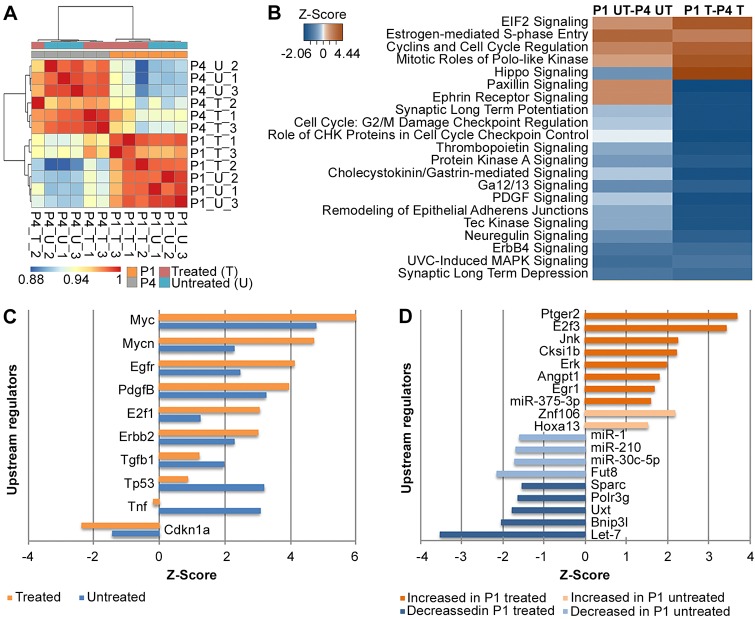
**Differential expression analysis of RNA-sequencing results from P1 and P4 *in vivo* samples after β-catenin stabilization.** (A) Pearson correlation heat-map demonstrates age as the main variability-causing factor following treatment. (B) IPA analysis of enriched signaling pathways. Z-score heat-map of significantly enriched signaling pathways comparing untreated samples at P1 and P4, and treated samples at P1 and P4. Orange represents pathway activation and blue represents pathway inhibition, *P*<0.005. (C) IPA analysis of most significantly enriched upstream regulators in treated (orange) compared with untreated (blue) samples. Z-scores are plotted. Positive and negative values indicate overall activation or inhibition of downstream targets, respectively, *P*<0.00001. (D) Mutually exclusive upstream regulators between treated and untreated comparisons as described for C, *P*<0.005. P1 samples were treated at P1 and harvested at P4; P4 samples were treated at P4 and harvested at P7.

Altogether, 12 samples were treated with tamoxifen and subjected to RNA-sequencing, including six *Sox2-CreER; β-catenin^flox(Exon3)/+^* pups at P1 or P4 (‘P1 or P4 treated’) and six Cre negative controls (‘P1 or P4 untreated/control’). Principal component analysis (PCA) outlined age as being responsible for 42% of the variation between samples, whereas treatment accounted for 17% of the variation. Pearson correlation heat-map of normalized counts was used to visualize the variability between samples. Samples were segregated by age and by treatment, presumably owing to differences in Cre activation between samples (Fig. 7A). Additional data analysis revealed insufficient Cre activation in one sample, apparent by the absence of exon 3 excision in *Ctnnb1* transcripts (Fig. S3). We therefore chose to exclude this sample from further analysis.

As the samples after β-catenin stabilization and the controls were genetically different, we first compared differentially expressed genes between P1 and P4 with and without β-catenin stabilization. 2914 genes were significantly differentially expressed in untreated samples at P1 compared with P4 and 3022 genes were significantly differentially expressed between P1- and P4-treated samples. Ingenuity pathway analysis (IPA; Qiagen) (Kramer et al., 2014) was subsequently performed to elucidate altered pathways in these datasets. Pathways related to cell proliferation, cell cycle, and cellular growth and development were enriched at P1 compared with P4, both with and without β-catenin stabilization. Nevertheless, β-catenin stabilization resulted in more robust and significant changes in the expression of these genes (Fig. 7B).

We next analyzed potentially enriched upstream regulators, whereby IPA clusters differentially expressed genes that are targets of a common regulator and observed further contribution of treatment to differential expression of proliferation-associated genes between P1 and P4. Higher activation and inhibition was observed for the 10 most significantly enriched regulators after treatment (Fig. 7C). Additionally, we present regulators that were enriched exclusively after treatment compared with the untreated samples (Fig. 7D). This suggests that the native expression differences, as well as the upregulation of cell-cycle and proliferation factors by stabilized β-catenin, underlie at least part of the regenerative capacity at P1 compared with P4.

### Stabilization of β-catenin alters expression of Wnt and Notch pathway genes at both P1 and P4

We evaluated the impact of β-catenin stabilization at each of the two time-points separately, while comparing the outcome between the two time-points thereby excluding genetic variability. After treatment at P1, 530 genes were significantly differentially expressed, with 240 increased and 290 decreased (median fold change=1.93). After treatment at P4, 304 genes were significantly differentially expressed, with 166 increased and 138 decreased (median fold change=1.73). In order to examine the effects of β-catenin stabilization, we first assessed Wnt signaling components using a list of Wnt pathway genes and targets from the RT² Profiler PCR Array (Wnt Pathway) and compared them with our *in vitro* results. Owing to the larger dataset available from RNA sequencing, we expanded the list used in the *in vitro* experiment to include additional Wnt components as well as targets from published sources (Hödar et al., 2010; Railo et al., 2009; Watanabe et al., 2014). At P1, 21 Wnt-associated genes were significantly increased and 10 were decreased after β-catenin stabilization, whereas at P4, 10 genes were significantly increased and four were decreased after treatment (Table 2). Six of the differentially expressed genes found here were also altered in our *in vitro* experiments, whereas at P4 only two genes were shared between the two systems. Nevertheless, both at P1 and P4, β-catenin stabilization led to Wnt activation, demonstrating that reduced regenerative capacity was not caused by an inability to activate Wnt.

**Table 2.**
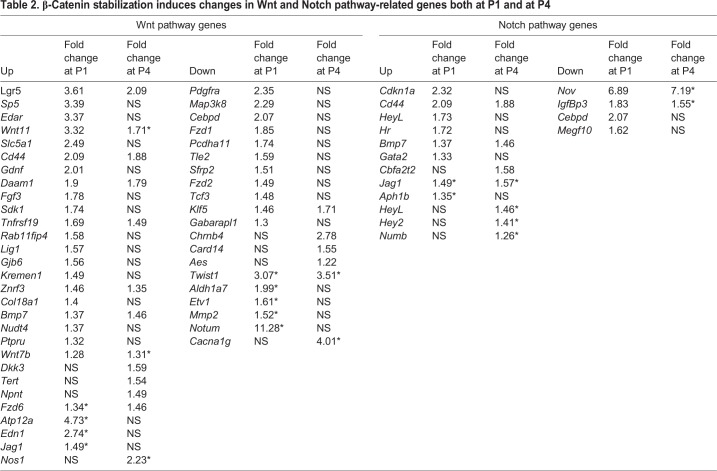
**β-Catenin stabilization induces changes in Wnt and Notch pathway-related genes both at P1 and at P4**

**Fig. 8.**
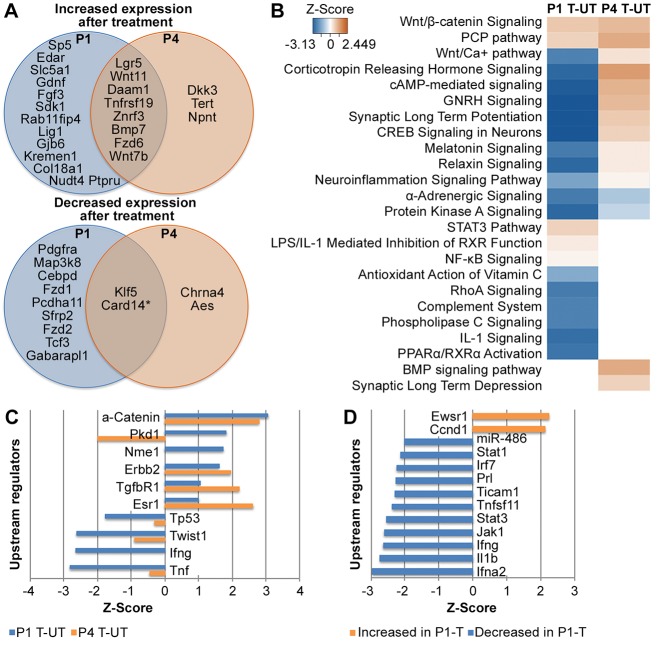
**Differential expression analysis of RNA-sequencing results comparing P1 and P4 *in vivo* samples with and without β-catenin stabilization.** (A) Venn diagram representing overlap between differentially expressed Wnt-related genes at P1 and P4. The overlap is more prominent in activated rather than inhibited genes. Genes that are marked in bold are more significantly altered at P1; genes that are marked with an asterisk are more significantly altered at P4 (*P*-adjusted <0.1). (B) IPA analysis identifies Wnt signaling as the most enriched pathway at both P1 and P4 after treatment. Positive Z-score (orange) represents activated pathways, negative Z-score (blue) represents inhibited pathways and non-enriched pathways are marked in white, *P*<0.05. (C) IPA analysis of most significantly enriched upstream regulators in P1 and P4 samples independently. Z-score presented for P4 (orange) and P1 (blue) comparisons, where positive and negative values indicate overall activation and inhibition of downstream targets, respectively, *P*<0.00001. (D) Mutually exclusive upstream regulators between treated and untreated comparisons, as described for C. No regulators were identified at P4 that were not enriched at P1, *P*<0.005. P1 samples were treated at P1 and harvested at P4; P4 samples were treated at P4 and harvested at P7.

The expression of several Wnt target genes was also altered, the most prominent of which was *Lgr5*, which encodes an R-spondin receptor. *Lgr5* is a Wnt target gene and a Wnt activator that is associated with SC regeneration in the inner ear (de Lau et al., 2014; Shi et al., 2012). Additional targets included *Znrf3*, *Tnfrsf19* and *Edar*, as well as the Notch-pathway associated genes *Cd44*, *Bmp7* and *Aes.* Overall, eight of the target genes that were upregulated after treatment were shared between P1 and P4 (Fig. 8A), and two of the genes that were downregulated after treatment were shared between P1 and P4.

We examined changes in the Notch pathway following β-catenin stabilization as well, using the list of genes from the RT² Profiler PCR Array (Notch Pathway) and supplementing it with MGI Gene Ontology database (GO:0005112, GO:0007219, GO:0007220, GO:0007221, GO:0045746 and GO:00457467). Out of 113, 96 Notch-pathway associated genes remained expressed in P4-untreated samples (normalized counts are in Table S1). Levels of the Notch target gene *Cdkn1a* were significantly increased *in vitro* and at P1 (Table 2) along with the Notch ligand *Jag1*, levels of which were also increased *in vitro* and at both P1 and P4. The activation indicators *Cd44* and *Heyl* were also increased at P4. Interestingly, expression of the transcriptional repressor *Hey2* was increased *in vitro*, but only at P4 *in vivo*.

An additional comparison of P1- and P4-treated samples pointed out general modification of the Wnt and Notch pathways between early and late neonatal stages that are further induced by β-catenin stabilization. Forty-eight Wnt-associated genes were increased and 39 were decreased at P1 compared with P4 (Table 3), whereas six Notch-associated genes were increased and 15 were decreased. Four additional Wnt-associated genes and two Notch-associated genes that were altered *in vitro* were significantly differentially expressed between P1 and P4. In summary, both the Wnt and Notch pathways are active in the maturing cochlea. Importantly, genes that are detected in P1-untreated samples can still be detected in P4-untreated samples.

**Table 3.**
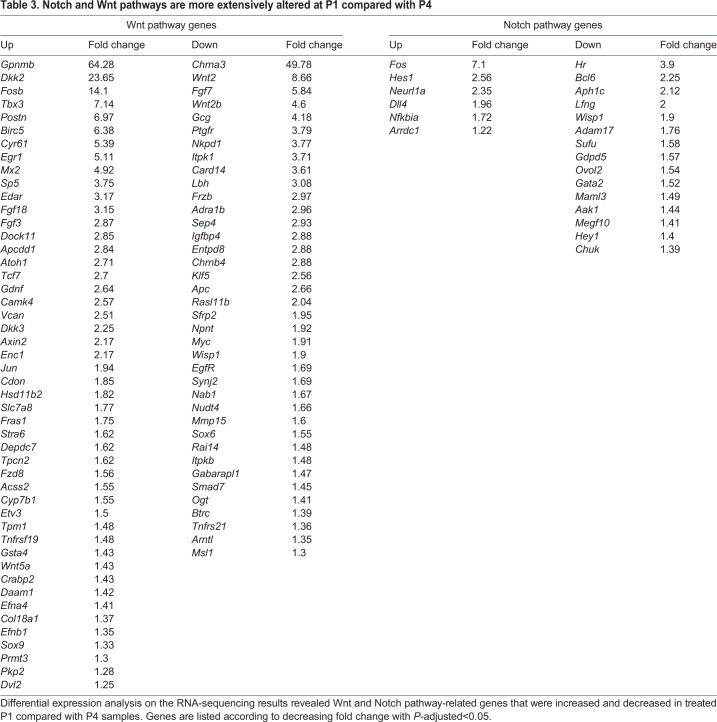
**Notch and Wnt pathways are more extensively altered at P1 compared with P4**

### Stabilization of β-catenin affects proliferation-related genes

IPA was further used to elucidate activated pathways. As expected, Wnt/β-catenin-signaling and planar cell polarity pathways were most prominently activated in treated samples (both at P1 and P4; Fig. 8B). Several maturation-related pathways were activated only at P4, whereas stress-related pathways were activated only at P1, suggesting different Wnt signaling downstream targets at the two time points. Wnt signaling, as well as the stress and proliferation pathway, were prominently enriched among upstream regulators after treatment at P1, whereas no such regulators were enriched at P4. *Notch1* was significantly enriched at P1 (Z-score=2.22, *P*=7.05E-6) and only weakly enriched at P4 (Z-score=1.35, *P*=0.04).

**Fig. 9.**
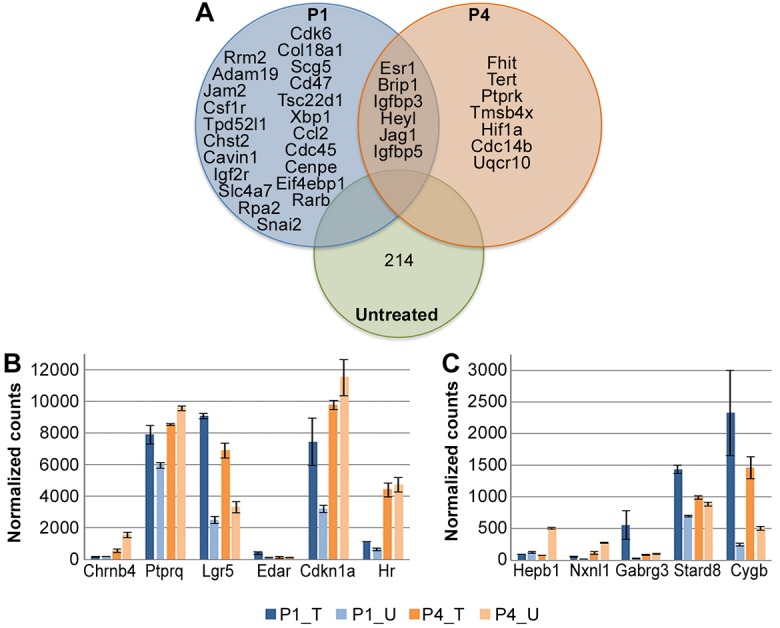
**Differential expression analysis of RNA-sequencing results demonstrating activation of *Erbb2* and additional factors after β-catenin stabilization.** (A) Venn diagram representing differential expression of *Erbb2* downstream targets at P1 and P4 in treated and untreated samples. 262 *Erbb2* targets are differentially expressed in untreated samples between P1 and P4, and 48 targets are also differentially expressed as a result of β-catenin stabilization, *P*<0.05. (B) Normalized counts for Wnt- and Notch-associated genes that present a significant pattern of differential expression relating to age and treatment simultaneously (*P*-adjusted <0.05). (C) Normalized counts for additional genes that present a significant pattern of differential expression relating to age and treatment simultaneously (*P*-adjusted <0.05). P1 samples were treated at P1 and harvested at P4; P4 samples were treated at P4 and harvested at P7.

The receptor tyrosine kinase gene *Erbb2*, which induces proliferation of several cell types (Abbas and Dutta, 2009), was detected as a significantly enriched upstream regulator in all comparisons (Figs 7C and 8C). As *Erbb2* levels were also increased after CHIR treatment in explants, we examined its pathway components. Overall, 262 *Erbb2* targets were differentially expressed in untreated samples at P1 compared with P4, in support of proliferation-associated signaling at early postnatal stages (Table S2). Nevertheless, when observing the differences at each time-point, 66 *Erbb2* targets were differentially expressed in P1-treated samples and only 29 at P4 (Table 4). Of interest is the overlap between *Erbb2* targets at P1 and P4, which are mostly associated with the Notch signaling pathway (Fig. 9A). The *Erbb2* target *Esr1* was detected as the most significantly differentially expressed gene in both P1- and P4-treated samples (fold change=13.68, *P*-adjusted=1.34E-50; fold change=14.71, *P*-adjusted=5.17E-48, respectively). It was also defined as a significantly enriched upstream regulator by itself at both time-points and estrogen-receptor signaling was among the activated pathways between P1 and P4 (Fig. 7B).

**Table 4.**
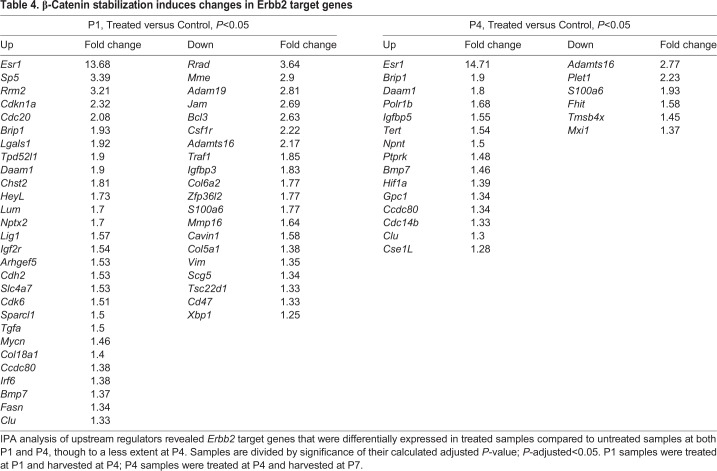
**β-Catenin stabilization induces changes in Erbb2 target genes**

Our intent was to identify processes, occurring after Wnt activation at P1 but not at P4, and thus potentially underlying the higher regenerative capacity at early neonatal stages. We therefore conducted a supplemental analysis and identified genes with a specific combined age and treatment interaction. The Wnt-pathway associated genes *Chrnb4*, *Ptpru*, *Lgr5* and *Edar*, the Wnt and Notch target gene *Cdkn1a*, and the Notch-pathway associated transcriptional repressor *Hr* had distinct and significant expression patterns (Fig. 9B). *Lgr5* was the only gene detected for which the expressional change appeared to result only from the treatment. *Edar* expression was influenced by treatment only at P1. *Chrnb4*, *Ptpru*, *Cdkn1a* and *Hr* expression increased with age in untreated samples. A small increase in the expression of negative regulators *Ptpru*, *Cdkn1a* and *Hr* was observed at P1, but not at P4. *Chrnb4* levels, however, decreased upon treatment at P4, with no effect at P1. In addition, the oxidative stress suppressors *Hebp1*, *Nxnl1* and *Cygb*, the tumor suppressor *Stard8* and the neuronal inhibitor GABA subunit *Gabrg3* were differentially expressed in an age- and treatment-dependent manner (Fig. 9C).

## DISCUSSION

**Fig. 10.**
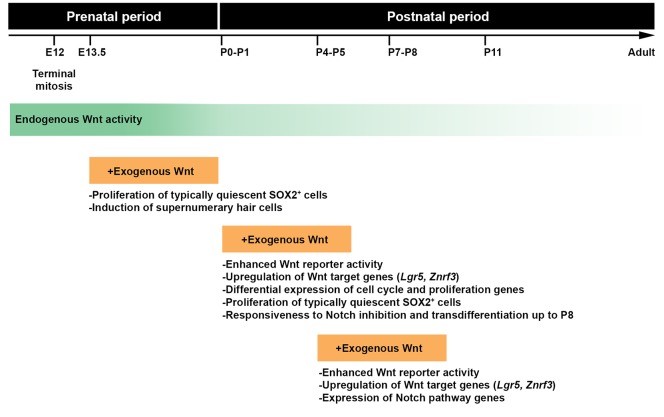
**Canonical Wnt pathway and the regenerative response.** A schematic representation of our working model. Canonical Wnt is highly active in the embryonic cochlea and, although mildly downregulated with age, Wnt pathway components continue to be expressed throughout neonatal stages and in the adult. However, downstream targets that are related to cell proliferation, cell-cycle progression and cellular growth are enriched at early neonatal stages compared with late stages, both with and without exogenous Wnt activation. This downstream transcriptional response to exogenous Wnt stimulation may therefore play a role in the age-dependent decline in the regenerative capacity of the postnatal mammalian cochlea.

In the developing mammalian cochlea, canonical Wnt is essential for prosensory cell proliferation and subsequent HC differentiation (Jacques et al., 2012; Shi et al., 2014). Forced Wnt activation in the neonatal cochlea promotes the proliferation of typically quiescent SCs and induction of ectopic HCs (Chai et al., 2012; Shi et al., 2013, 2012). However, in contrast to earlier developmental stages, the adult mammalian auditory organ lacks capacity for HC regeneration in response to Wnt (Shi et al., 2013). Here, using both *in vitro* and *in vivo* models, we describe the temporal pattern and the underlying mechanisms of the regenerative response to Wnt activation in the embryonic and neonatal mouse cochlea (Fig. 10).

Stimulation with CHIR *in vitro* at E13.5 and P0 causes prosensory cells and SCs, respectively, to re-enter the cell cycle. At P0, the position of SCs along the basal-apical axis has a clear effect on the strength of the proliferative response, with a greater number of proliferated SCs observed in the apex compared with the base. Owing to the basal-apical developmental gradient (Chen et al., 2002), SCs in the apex are likely to be less mature than those in the base at birth. Furthermore, we demonstrate that the proliferative response to Wnt is no longer observed by P5, presumably owing to SC maturation. This type of transient regenerative response is also observed in the mouse heart and retina (Jorstad et al., 2017; Porrello et al., 2011).

In organs that regenerate in the adult mammal, like gut, skin and liver, stemness is maintained by active Wnt signaling (Kessler et al., 2015). In the intestines, for example, depletion of β-catenin and resultant loss of Wnt signaling leads to the rapid loss of stem cells and capacity for self-renewal (Fevr et al., 2007). Here, using western blots we demonstrate that the age-dependent decline in proliferative capacity in the cochlea does not correlate with diminished β-catenin accumulation in the older (P5) stage. In fact, compared with controls, exposure to CHIR promoted similar levels of β-catenin accumulation at all three developmental stages. It could also be argued that the dampened nature of the proliferative response with age could correlate with an upregulation of components that inhibit canonical Wnt signaling in the older cochlea (Hirsch et al., 2007). In such an instant, Wnt-reporter activity at P5 should remain unchanged following exposure to the Wnt activator. In contrast, we show that stimulation with CHIR enhances TCF/Lef complex activation at P5, similar to its effect at P0. Furthermore, we find that canonical Wnt signaling is active in a subset of SCs, as well as HCs at both time points. This is in agreement with our recent findings showing that Wnt pathway components continue to be expressed in the mouse cochlea even at P30 (Geng et al., 2016) and is further supported by our RNA-sequencing results from *in vivo* Wnt activation. It is likely that the Wnt pathway plays a role in homeostatic maintenance of the cochlea; however, future loss-of-function experiments are needed to fully elucidate its roles in the adult.

CHIR is a potent activator of canonical Wnt signaling. It was therefore administered at several different concentrations, depending on the age of the culture and expected response to Wnt in terms of proliferation and HC induction. For evaluation of the *TCF/Lef:H2B-GFP* neonatal response, a shorter treatment at lower concentration was needed to prevent saturation. Non-canonical Wnt signaling is also involved in the cell-adhesion processes, which may influence the status of TCF/Lef activation when the cochlear explants adhere to the culture dish (Gallegos et al., 2016; Heuberger and Birchmeier, 2010). We therefore limited the incubation time to exclude potential interference and masking of canonical Wnt activation.

Similar to the age-dependent decline in the proliferative response to Wnt, Maass et al. (Maass et al., 2015) previously demonstrated that SCs fail to transdifferentiate to HCs following Notch inhibition by P3. However, we demonstrate that augmenting Wnt activation through the neonatal stages extends this window for HC induction in response to Notch inhibition up to at least P8. Comparable with that observed by Ni et al. (Ni et al., 2016a,b), we find that the combination of Wnt activation followed by Notch inhibition not only generates new HCs but also replenishes the SC pool, both of which are necessary for proper auditory function. In combination with reports on development and regeneration in the mammalian cochlea (Bramhall et al., 2014; Jayasena et al., 2008; Maass et al., 2015), our results suggest that Wnt signaling modulates the Notch pathway during these processes. Intriguingly, stimulation with CHIR resulted in the downregulation of Notch receptors but promoted a significant upregulation of the Notch ligand and SC marker *Jag1*. This downregulation of Notch receptors may represent a negative-feedback mechanism to limit high levels of Notch signaling resulting from crosstalk with the Wnt pathway (Collu et al., 2014). We hypothesize that newly proliferated SCs remain responsive to Notch signaling inhibition up to P8, as they represent immature cells that exhibit prosensory character.

Although SCs proliferate and form immature prosensory-like cells in response to Wnt activation at P0, this proliferative response is not observed by P5. Our previous results *in vivo* (Shi et al., 2013), together with our current *in vitro* analysis, led us to use RNA sequencing to look for explanations for this difference in response to Wnt. Indeed, we have demonstrated here that the age-dependent decline in proliferative capacity is correlated with changes in the downstream transcriptional response to Wnt activation. Although our RNA analysis showed that both Wnt and Notch pathway components continue to be expressed throughout early and late neonatal stages, downstream targets that are related to cell proliferation, cell-cycle progression and cellular growth were enriched at P1 compared with P4, both with and without β-catenin stabilization. Most prominently, the proliferation-associated gene *Esr* and the upstream regulator *Erbb2* are demonstrated here with significant differential enrichment between early and late neonatal stages. Specifically in the neonatal inner ear, *Erbb2* is expressed in pillar, Hensen's and Claudius cells, and weakly in the greater epithelial ridge (GER) (Hume et al., 2003). These EGFR^+^ SCs were identified with extended proliferative capacity, overlapping with an EGFR-dependent regeneration mechanism from birds (White et al., 2012). Pillar and GER cells are also Lgr5 positive, thus possessing extensive proliferative traits (Shi et al., 2012). Although *Erbb2* itself was mildly downregulated after CHIR treatment, its downstream components are activated following β-catenin stabilization.

Additional factors were also differentially expressed in a combined, age- and treatment-dependent manner. *Hebp1* suppresses oxidative stress as part of the Nrf2 pathway and can protect against cisplatin and noise-induced hearing loss (Honkura et al., 2016; Kim et al., 2015). Its normal expression appeared to increase with age but Wnt activation decreased its expression at P1 and even more significantly at P4. *Nxnl1*, a viability factor in eye development that is associated with retinitis pigmentosa and Bardet-Biedl syndrome (Bin et al., 2009; Mei et al., 2016), is also involved in suppression of oxidative stress and possessed a similar pattern of expression. These two genes are associated with protection of post-mitotic cells. Their expression is unchanged after treatment at P1, presumably owing to their already low expression, but is decreased after treatment at P4, which could reflect epigenetic alterations to a less mature state as an attempt to regain proliferation capabilities. In contrast, the neuronal inhibitor GABA subunit *Gabrg3*, which is expressed in outer and inner HCs (Girotto et al., 2014), and tumor suppressor *Stard8* were significantly upregulated after Wnt activation at P1 but not at P4, suggesting a potential feedback mechanism to repress proliferation. Additional pathways downstream of Wnt may therefore underlie the reduced proliferative capacity observed at late postnatal stages, presumably silenced by epigenetic regulation. Indeed, we have recently demonstrated that the proliferative response of SCs is improved when cultured with a Wnt activator and a histone deacetylase inhibitor (McLean et al., 2017).

We have shown previously that SOX2 levels influence Wnt and Notch-induced proliferation and differentiation of cochlear cells (Atkinson et al., 2018; Jeon et al., 2011; Kempfle et al., 2016). One limitation of the approach used here is the reduced *Sox2* levels in the *Sox2* knock-in alleles such as *Sox2-CreER* and *Sox2-GFP*, and the direct comparisons between drug treatment *in vitro* and genetic manipulation *in vivo* (Arnold et al., 2011; Bramhall et al., 2014). Our *in vitro* results, obtained here from *Sox2-GFP* mice, agree with the results of similar experiments that were conducted on wild-type mice, and we found a similar expression pattern of Wnt signaling genes *Wnt11*, *Fzd2* and *Tcf3* between β-catenin stabilization (*in vivo*) and CHIR-treated (*in vitro*) samples. The Notch target gene *Cdkn1a* was also similarly expressed. Distinctions between the expression of additional Wnt and Notch components are most likely due to differences between *in vivo* and *in vitro* experiments, as well as *in vivo* β-catenin stabilization compared with the use of a GSK3β inhibitor.

In summary, we show that exogenous Wnt activation following birth not only promotes the proliferation of SCs but also extends the window for HC induction up to P8, thus generating new HCs in the maturing cochlea. Exogenous Wnt activation potentially promotes an arrest of maturation progression or even partial dedifferentiation of SCs into a less mature state. Our results suggest that efforts to stimulate regeneration in the adult auditory organ may require recapitulating the transcriptional response of the early neonatal cochlea to exogenous Wnt activation and subsequent alterations of silenced state of Wnt target genes.

## MATERIALS AND METHODS

### Mice

Male and female CD-1 mice (Charles River) and *Sox2^EGFP/+^* reporter mice (Jackson Laboratories, strain B6;129S-Sox2^tm2Hoch^/J, stock number 017592) (Arnold et al., 2011) were maintained and euthanized in accordance with Institutional Animal Care and Use Committee regulations at Sunnybrook Research Institute, Toronto, Canada.

Male and female *Sox2-CreER; β-catenin^flox(Exon3)/+^* mice were used to stabilize β-catenin in SOX2^+^ cells. *Sox2-CreER* (Arnold et al., 2011) and *β-catenin^flox(Exon3)/+^* (Harada et al., 1999) were kindly provided by K. Hochedlinger (Harvard Medical School, Boston, MA, USA) and M. Taketo (Kyoto University, Kyoto, Japan), respectively. *TCF/Lef:H2B-GFP* Wnt-reporter mice [Jackson Laboratories, strain Tg(TCF/Lef1-HIST1H2BB/EGFP)61Hadj/J, stock number 013752] were used to visualize Wnt activation. These strains were maintained and euthanized in accordance with Institutional Animal Care and Use Committee regulations at the Massachusetts Eye and Ear Infirmary.

### Organotypic cultures

Cochlear ducts were dissected from embryos of timed-pregnant mice at embryonic day (E) 13.5 and neonatal pups at P0 and P5, and the roof of the duct was removed to expose the sensory epithelium. Cochleae were established as explant cultures as previously described (Mulvaney et al., 2013). The Wnt pathway was activated with CHIR99021 (1.5 μM at E13.5; 5 or 10 μM at P0 and P5; EMD Millipore) for 5 or 8 days *in vitro* (DIV). Cochlear explants of littermates were cultured with vehicle (DMSO) as controls. Proliferation under both conditions was assayed by supplementing culture media with BrdU (3.5 μg/ml; BD Biosciences). The Notch pathway was inhibited with DAPT (25 μM; EMD Millipore). Each experiment was performed with a minimum of three independent litters and a minimum of seven cochleae per condition.

In order to visualize Wnt activation, cochlear explant cultures of *TCF/Lef:H2B-GFP* reporter mice were established at P0 and P5 and incubated with CHIR99021 (3 μM; Cayman Chemicals) for 13 h. Cochlear explants from littermates were cultured with DMSO as controls.

### Live imaging

Cochlear explant cultures from E13.5 *Sox2^EGFP/+^* reporter mice were established and imaged as previously described using an Olympus VivaView FL Incubator Microscope (Mulvaney and Dabdoub, 2014). The Wnt pathway was activated with CHIR99021 (1.5 μM; EMD Millipore) for 5 DIV. Cochlear explants of littermates were cultured with DMSO as a control.

### Immunofluorescence

Immunofluorescence detection was performed as previously described (Jacques et al., 2012). The following primary antibodies were used: goat anti-SOX2 (1:250; Santa Cruz Biotechnology, sc-17320), rabbit anti-MYOVI (1:1000; Proteus BioSciences, 25-6791), rabbit anti-MYOVIIA (1:1000; Proteus BioSciences, 25-6790), goat anti-JAG1 (1:250; Santa Cruz Biotechnology, sc-6011), rat anti-E-cadherin (1:500; Abcam, ab11512), rabbit anti-cyclin D1 (1:250; Thermo Fisher Scientific, MA5-14512), mouse anti-BrdU (1:250; BD Biosciences, 555627) and chicken anti-GFP (1:500; Abcam, ab13970). Prior to BrdU staining, antigen retrieval was performed with 1 N HCl for 30 min. Images were captured using a Nikon A1 Laser Scanning Confocal Microscope or a Leica SP8 Laser Scanning Confocal Microscope. Images of samples from *TCF/Lef:H2B-GFP* mice were prepared using a *z*-projection from a stack covering the entire depth of the explant. The top segment (7.25 μm thick) was defined as the HC layer according to apparent MYOVIIA expression in the image annotation; the bottom segment (4.75 μm thick) was defined as the SC layer according visualized SOX2. Samples from a specific time-point were harvested, treated and analyzed simultaneously, and the GFP signal gain was determined using the control sample and then applied to the treated sample.

### Cell quantification

Proliferation in E13.5 cochlear explants treated with control media or CHIR99021 was assessed by calculating the percentage of SOX2^+^BrdU^+^ cells among all SOX2^+^ cells within a 200×200 μm box positioned over the HC domain, at the 50% position from the base of cochlea. The number of differentiated HCs in E13.5 explants were assessed by counting MYOVIIA^+^ cells within a 200 μm segment along the length of the cochlea and the entire width of the sensory epithelium at the 50% position from the base of the cochlea. Proliferation in P0 and P5 cochlear explants treated with control media or CHIR99021 was assessed by calculating the percentage of SOX2^+^BrdU^+^ cells among all SOX2^+^ cells within a 200 μm segment along the length of the cochlea and the entire width of the sensory epithelium at positions 25%, 50% and 75% from the base of cochlea. HC induction in response to Notch inhibition in control or CHIR99021-treated samples was assessed by counting MYOVI^+^BrdU^+^ cells per 200 μm segment along the length of the cochlea and the entire width of the sensory epithelium at positions 25%, 50% and 75% from the base of cochlea. Cell counts for each experiment were obtained from a minimum of three independent litters and a minimum of seven cochleae per condition. Statistics were performed using a two-tailed Student's *t*-test with the significance threshold set to *P*<0.05.

### Western blots

Similar to the procedure used for cochlear explant cultures, cochlear ducts were dissected from E13.5, P0 and P5 mice, and the roof of the duct was removed to expose the sensory epithelium. Cochleae were subsequently exposed to 10 μM CHIR99021 or DMSO in DMEM for 3 h. Each experiment was performed with a minimum of three independent litters and six cochleae per condition. Cochleae were lysed in chilled lysis buffer [150 mM sodium chloride, 1% NP-40, 50 mM Tris (pH 8.0)]. Lysate was centrifuged at 14,000 ***g*** for 20 min at 4°C and supernatant was collected. Proteins were separated using Bolt Mini Gels (Life Technologies) according to the manufacturer's instructions. Proteins were blotted onto polyvinylidene difluoride membranes and immunoblotted as previously described (Mulvaney et al., 2015). The following primary antibodies were used: mouse anti-active β-catenin (0.5 µg/ml; EMD Millipore, 05-665), mouse anti-β-catenin (1:2000; BD Biosciences, 610153) and mouse anti-GAPDH (1 µg/ml; Applied Biosystems, AM4300). Imaging and densitometric analyses were performed using the ChemiDoc MP Imaging System and Image Lab Software (Bio-Rad). Statistical analyses were carried out using a two-tailed Student's *t*-test with the significance threshold set to *P*<0.05.

### RT^2^-profiler PCR arrays

Cochlear explants established on P0 were cultured with DMSO or 10 μM CHIR99021 for 5 DIV. RNA was extracted from three independent litters and five cochleae per condition using RNeasy Mini Kit (Qiagen) according to manufacturer's instructions. Concentration of RNA was determined using a Qubit Fluorometer (Thermo Fisher Scientific) and cDNA was prepared using the RT^2^ First Strand Kit (Qiagen). Quality of reverse transcription reaction was assessed with RT² PCR Array Mouse RNA QC plates (Qiagen, PAMM-999Z). Amplification of genomic DNA was not detected. PCR was performed according to manufacturer's instructions using RT² SYBR Green ROX qPCR Mastermix (Qiagen) and RT² Profiler PCR Array Mouse Wnt Signaling Pathway (Qiagen, PAMM-043ZC) or RT^2^ Profiler PCR Array Mouse Notch Signaling pathway (Qiagen, PAMM-059ZC). These arrays were pre-designed to detect 84 Wnt pathway genes and 84 Notch pathway genes, respectively. Thermal cycling was performed using StepOnePlus Real-Time PCR system (Applied Biosystems) as previously described (Geng et al., 2016). Quality control, fold changes in gene expression and statistical analysis (two-tailed Student's *t*-test with the significance threshold set to *P*<0.05) was performed using RT^2^ Profiler PCR Array Data Analysis Workbook, Version 4 (Qiagen, www.qiagen.com/ca/resources/resourcedetail?id=d8d1813e-e5ba-4d29-8fdf-07a3f4227e0a&lang=en). A complete list of genes assessed can be accessed through Qiagen for both RT² Profiler PCR Array Mouse Wnt Signaling Pathway (www.qiagen.com/ca/shop/pcr/primer-sets/rt2-profiler-pcr-arrays/?catno=PAMM-043Z#geneglobe) and RT^2^ Profiler PCR Array Mouse Notch Signaling pathway (www.qiagen.com/ca/shop/pcr/primer-sets/rt2-profiler-pcr-arrays/?catno=PAMM-059Z#geneglobe).

### Real-time quantitative PCR (RT-qPCR)

RNA was prepared as described above and cDNA was transcribed using the High Capacity RNA-to-cDNA Kit (Applied Biosystems). The following primers (Applied Biosystems) were used for RT-qPCR gene expression assays: *Gapdh* (Mm99999915_g1) as control, *Jag1* (Mm00496902_m1), *Jag2* (Mm01325629_m1), *Notch1* (Mm00435249_m1), *Notch2* (Mm00803077_m1), *Notch3* (Mm01345646_m1), *Notch4* (Mm00440525_m1), *Fzd4* (Mm00433382_m1) and *Wnt11* (Mm00437328_m1). Statistics were obtained using a two-tailed Student's *t*-test with the significance threshold set to *P*<0.05.

For H2B-GFP analysis, cDNA was transcribed using the qScript XLT cDNA Synthesis Kit (QuantaBio) and qPCR was conducted using SYBR Select SYBR Mix (Applied Biosystems) with Gapdh PrimePCR SYBR Green Assay (Bio-Rad) and the following primers for H2B-GFP: forward, CAAGATCCGCCACAACATCG; reverse, GACTGGGTGCTCAGGTAGTG. Statistical analyses were carried out using a two-tailed Student's *t*-test with the significance threshold set to *P*<0.05.

### Stabilization of β-catenin *in vivo*, RNA extraction and sequencing

For Cre activation in neonatal mice, mothers were given 100 μl tamoxifen (50 μg/μl, Sigma-Aldrich) via intraperitoneal injection 1 or 4 days after delivery. Thus, *Sox2-CreER; β-catenin^flox(Exon3)/+^* mice were exposed to tamoxifen at P1 or P4 through the mother's milk and sacrificed 72 h later at P4 or P7, respectively. To verify Cre activation, brain tissue was collected from neonatal mice and genotyped using Kapa Mouse Genotyping Kit (Kapa Biosystems) with the following primers: forward, GGT AGG TGA AGC TCA GCG CAG AGC; and reverse, ACG TGT GGC AAG TTC CGC GTC ATC C. PCR amplified samples were separated on a 1% agarose gel. A 900 bp band was used to identify *Ctnnb1* wild-type allele and a 700 bp band was used to identify the exon3 excision. Littermates carrying *β-catenin^flox(Exon3)/+^* but without *Sox2-CreER* were used as controls.

Once dissected, cochleae were incubated in Cell Recovery Solution (Corning) for 45 min to separate HCs and SCs from the underlying mesenchyme and neurons. RNA was extracted using RNeasy Micro Kit (Qiagen) from three individual mice per condition, separately and without pooling.

Library preparation and RNA sequencing was performed at the Dana Farber Cancer Institute Molecular Biology Core Facility. In brief, cDNA was synthesized from 2.5 ng of RNA using SMARTer V4 Kit (Clontech). Following fragmentation using M220 Focused-Ultrasonicator (Covaris), 2 ng of sheared cDNA was taken for library preparation using ThruPLEX DNA-seq kit (Rubicon Genomics). NextSeq500 Single-End 75 bp (SE75) Sequencing (Illumina) was performed on all 12 samples in one lane after they were indexed and pooled in equimolar amounts to ensure 20-30 million reads per sample.

Reads were aligned to mm10 augmented with Ensembl gene build 75 with STAR v2.5.2b (Dobin et al., 2013). Reads and alignments were assessed for quality using a combination of FastQC (www.bioinformatics.babraham.ac.uk/projects/fastqc/), Qualimap (Garcia-Alcalde et al., 2012) and custom scripts. For quality control purposes, counts of reads per gene were generated using FeatureCounts (Liao et al., 2014). For quantification, transcripts per million (TPM) measurements were generated using Sailfish v0.10.1 (Patro et al., 2014). Transcript counts were collapsed to gene level counts using Tximport (Soneson et al., 2015). All 12 samples showed 40-50 million reads per sample. Above 90% of the genes aligned to the genome and over 23,000 genes were detected for each sample. Differential expression was evaluated using DESeq2 (Love et al., 2014), fitting a model accounting for treatment and age of the samples (data have been deposited in GEO under accession number GSE113719). Genes were called differentially expressed when the absolute value of the fold change was >1.25 and *P*-adjusted (FDR) (Benjamini and Hochberg, 1995) was smaller than 0.05 or 0.1, as mentioned. Subsequent pathway analysis was performed using Ingenuity Pathway Analysis software (Qiagen). Of note is the differential expression of the X-inactive specific transcript (*Xist*), which pointed out the potential bias that might occur due to the random segregation of female and male mice in our dataset (Fig. S4). We therefore excluded from our results any gene on chromosome X with the same pattern of differential expression as *Xist*, which could account from gender association rather than age or treatment.

## Supplementary Material

Supplementary information
