# Supplementary material for: Transcriptional response to Wnt activation regulates the regenerative capacity of the mammalian cochlea
Source: Development. 2018 Nov 27;145(23):dev166579. doi: 10.1242/dev.166579 (PMC6288390; doi:10.1242/dev.166579)
Supplement: Supplementary information [file develop-145-166579-s1.pdf]

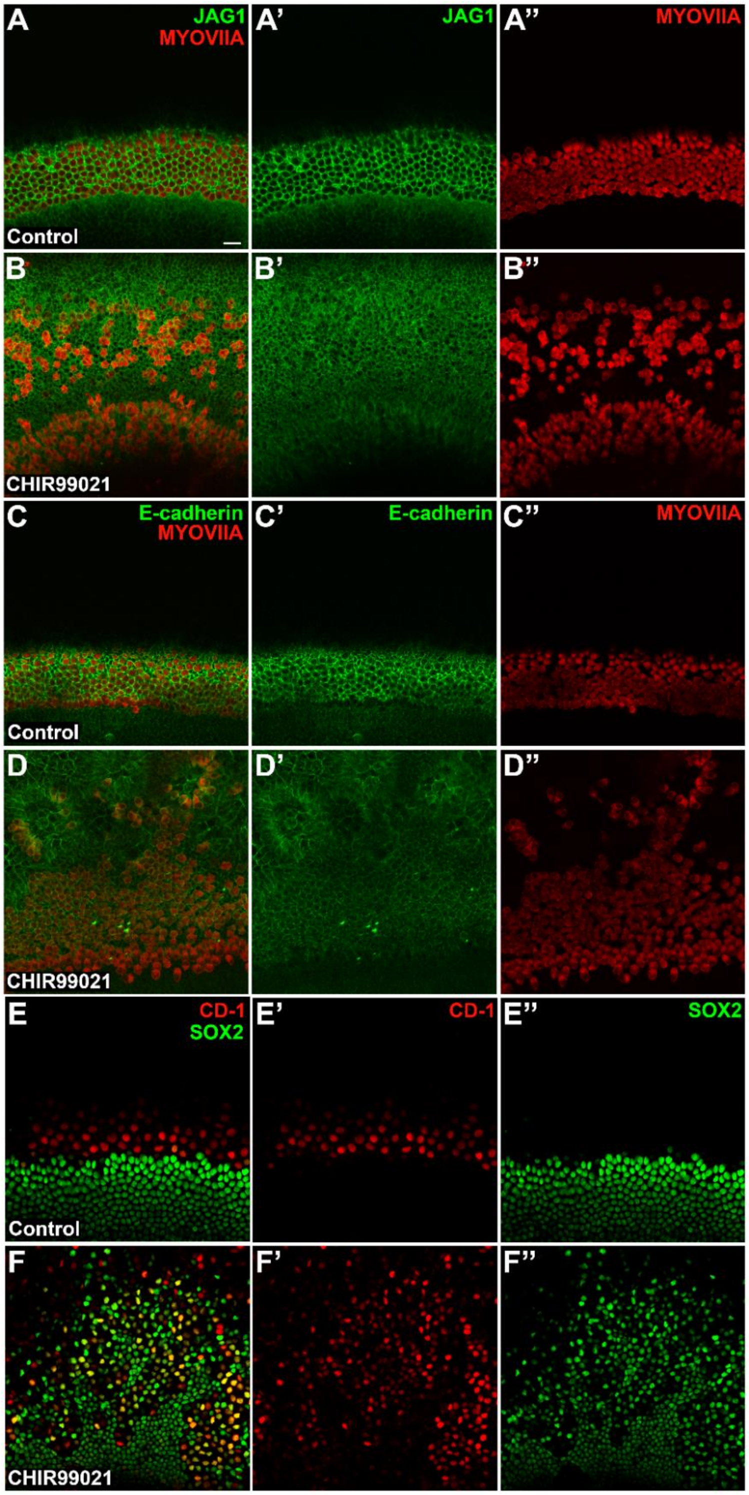

Figure S1. Wnt activation at E13.5 promotes cell cycle re-entry in SOX2-positive cells and a lateral expansion of the sensory epithelium.
(A-A") E13.5 cochlear explant cultured for 5 days with control media and immunolabelled for JAG1 (green) and MYOVIIA (red). (B-B") Wnt activation at E13.5 with CHIR for 5 DIV resulted in a lateral expansion of the $JAG1^+$ domain, compared to controls. (C-C") E13.5 cochlear explant cultured for 5 days with control media and immunolabelled for E-cadherin (green) and MYOVIIA (red). (D-D") Wnt activation at E13.5 with CHIR for 5 DIV resulted in a lateral expansion of the $E\text{-cadherin}^+$ domain, as well as reduced membrane localization. (E-E") E13.5 cochlear explant cultured for 5 days with control media shows immunostaining for $SOX2^+$ cells (blue) and CyclinD1 (red). CyclinD1 expression was not detected in $SOX2^+$ cells in control explants. (F-F") E13.5 cochlear explant cultured for 5 days with CHIR shows a substantial increase in $SOX2^+CyclinD1^+$ cells following Wnt activation. Scale bar: 20 μm in A-F.

Development • Supplementary information

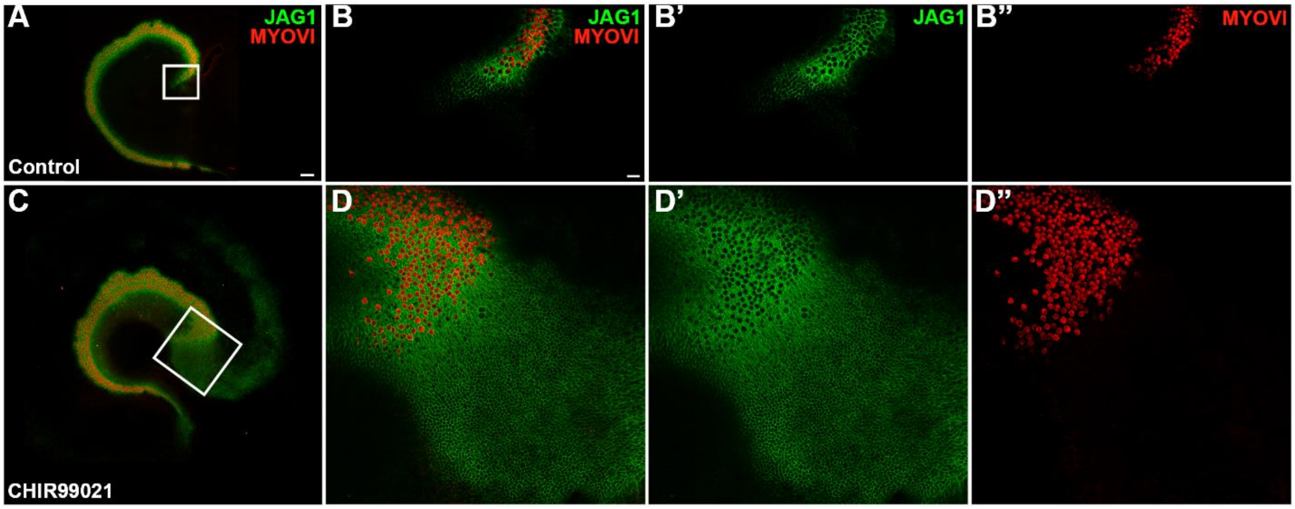

Figure S2. Wnt activation at E15.5 results in an expansion of JAG1-positive domain at the apex.
(A) Low-magnification view of cochlear explant established at E13.5 and cultured with control media at E15.5 for 4 days. (B-B") High-magnification view of apex of control explant shows immunostaining for JAG1 (green) and MYOVI (red). (C) Low-magnification view of cochlear explant established at E13.5 and cultured with CHIR on E15.5 for 4 days shows an expansion of the $JAG1^{+}$ domain at the apex. (D-D") High-magnification view of apex of CHIR treated explant shows an extended $JAG1^{+}$ domain and an increase in the number of differentiated hair cells. Scale bars: 100 µm in A, C; 20 µm in B, D.

Development • Supplementary information

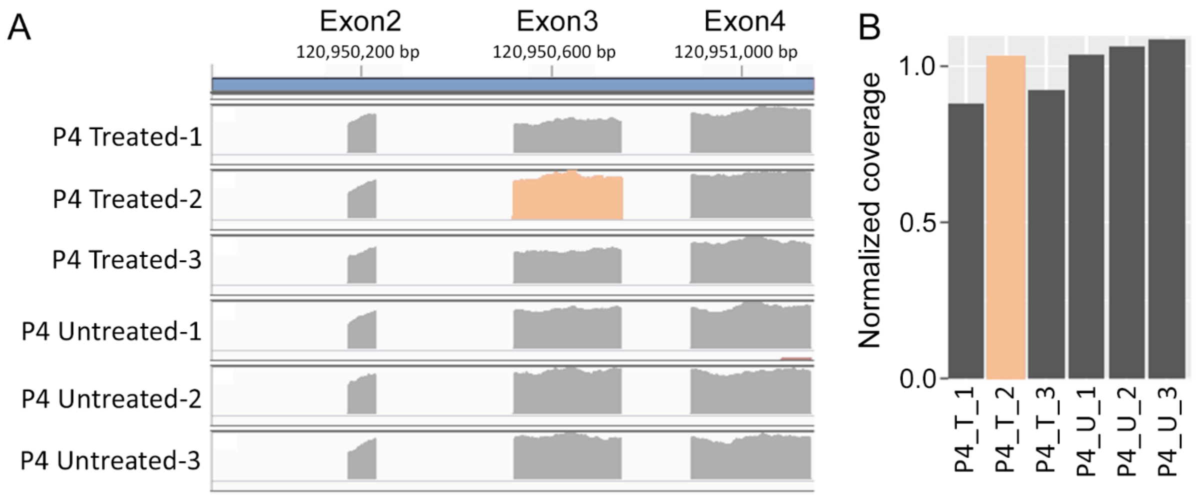

Figure S3. Assessment of Cre activation after tamoxifen treatment.
Ctnnb1 genomic location is enlarged to emphasize exons 2-4 (A) Normalized counts are presents for each sample from P4 treated and un-treated for each of the three exons (A) and for exon3 only (B) While exon3 levels are lower in treated samples 1 (P4_T_1) and 3 (P4_T_3), they are similar to untreated levels for sample 2 (P4_T_2), indicating insufficient Cre activation in this sample.

Development • Supplementary information

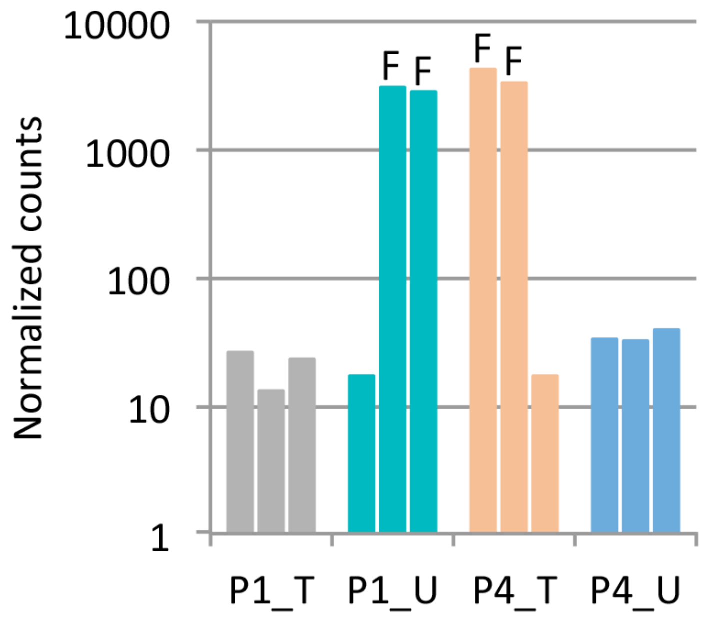

Figure S4. *Xist* expression defines uneven gender segregation in tested samples.
Normalized counts of X-inactive specific transcript (*Xist*), expressed in female mice, demonstrate that all 3 P1 treated and P4 control samples were from male mice, while 2 out of 3 P1 control and P4 treated samples were from female mice. The male sample from P4 treated sample is the sample that was not included in the analysis due to insufficient Cre activation, leading to almost complete gender segregation between treated and control samples. F marks samples from female mice.

Development • Supplementary information

Table S1. Notch-pathway associated genes are expressed at P4 and at P7.
Normalized counts of 113 Notch-pathway genes were examined, revealing reliable expression (average normalized counts > 100) of 96 genes that are expressed in both P1 and P4 control samples that are harvested at P4 and at P7, respectively.

Gene	P1_U_1	P1_U_2	P1_U_3	P4_U_1	P4_U_2	P4_U_3
Hr	579	829	559	4644	3989	5595
Cdkn1a	2758	3386	3481	10140	13830	10557
Lfng	2924	3616	2662	6759	8633	8754
Lmo2	190	138	172	409	431	427
Aph1c	545	502	559	1018	1150	1362
Bcl6	132	171	155	291	319	350
Wisp1	4537	5967	5591	7314	12282	11419
Runx1	147	213	278	394	291	471
Adam17	2649	2448	2786	5383	3621	5014
Gdpd5	6446	5732	5456	9857	9428	11758
Bend6	123	93	133	59	254	300
Gata2	18592	17875	18017	33357	28073	34106
Maml3	866	1043	1128	1656	1307	1959
Notch4	78	92	143	258	106	143
Aph1b	1860	2210	1690	2700	2983	3272
Gli1	52	117	83	121	132	122
Aak1	575	644	541	942	724	929
Sufu	1518	1589	1425	2230	1979	2465
Ovol2	542	369	555	622	701	822
Ncor2	2491	2313	2258	3386	3183	3435
Hif1an	2023	2026	1541	2985	2071	2811
Cd44	3434	3958	2768	4094	4560	5515
Hey1	7379	9507	7675	11544	10596	11341
Chuk	2707	2894	2462	3527	3475	3555
Id1	3932	5290	4872	6114	6226	5817
Nfkb1	428	513	462	528	658	578
Arrb1	386	265	327	426	353	448
Rita1	350	356	406	446	450	485

Development • Supplementary information

Ctnnb1	21583	25494	19992	25943	26551	30753
Supt6	1849	2186	1822	2548	2189	2505
Stat6	1224	1486	1082	1739	1291	1641
Ep300	1766	2033	1784	2272	1876	2614
Egfl7	1116	1149	1231	1545	1320	1351
Rbm15	240	253	180	273	278	245
Gsk3b	4770	4890	4958	4666	5891	6705
Cul3	5784	5519	5634	7333	5888	6516
Kctd10	3557	3831	3446	4303	3938	4381
Mmp14	21323	22436	21817	24681	23846	27427
Cbl	632	557	381	679	614	522
Nfkb2	685	614	534	722	635	762
Aph1a	1030	1184	1184	1140	1322	1465
Heyl	4222	5814	3877	5773	4659	5541
Notch1	8046	6392	6464	7896	5915	10146
Cflar	1984	1831	2043	2289	2029	2337
Erbb2	4602	4198	4736	5451	4203	5499
Ccnd1	2051	1520	1399	1389	2196	1904
Hey2	1361	1492	1201	1237	1614	1601
Lrp5	366	385	389	390	379	472
Rbpjl	121	114	91	133	92	128
Sel1l	10393	10443	9057	10959	9654	11418
Adam10	9434	9522	8864	11380	7894	10371
Maml1	434	453	507	449	554	479
Rfng	2668	3162	2919	2968	3146	3163
Psen1	4123	4609	4662	4892	4529	4725
Dner	729	761	910	729	812	973
Arrdc1	3755	3530	3784	4192	3459	3796
Igfbp3	33374	39537	30127	34944	33838	36794
Galnt11	3784	3392	3048	3114	3610	3528
Pofut1	1304	1333	1448	1128	1453	1505
Aes	16000	16558	17725	16776	16454	16159

Development • Supplementary information

Numb	3156	3127	2748	3030	2963	2794
Psenen	1945	1878	2173	1884	2075	1859
Ncstn	17295	18969	17183	16654	16648	18322
Psen2	764	739	711	651	774	712
Dll1	135	138	113	80	125	164
Mfng	1106	1342	1036	1204	1032	1007
Jag1	19133	21372	14022	19694	13254	17765
Snw1	5992	6382	6157	5445	5594	5866
Smo	5934	5713	5765	5286	4939	5417
Notch3	2794	2817	2777	2734	2091	2578
Nrarp	2044	2498	2226	2266	1866	1834
Tle1	5590	6675	6672	5860	5357	5436
Cbfa2t2	2325	2764	1812	2271	1851	1926
Krt1	114	116	136	106	124	84
Axin1	4176	4145	3900	3734	3069	3638
Slc35c1	1315	1354	1276	1150	1052	1160
Ccne1	151	205	238	169	185	148
Maml2	1267	1450	1507	1267	1258	1016
Nr4a2	696	858	866	757	532	679
Bmp7	11072	9606	9242	7204	7547	8992
Dlk2	1822	2189	1656	1345	1631	1407
megf10	911	1059	861	790	626	747
Il6st	3552	4193	3673	3497	2099	3126
Nfkbia	4392	5058	4879	3275	3827	3620
Dll4	317	316	202	246	203	165
Lor	261	266	255	195	223	136
Notch2	1244	964	1032	865	544	774
Dtx1	598	741	611	288	380	570
Zic2	134	149	198	72	179	49
Hes5	742	575	506	506	340	266
Jag2	2409	2170	1612	1192	1101	1320
Cebpd	357	488	436	176	237	249

Hes1	1671	2771	1795	908	1029	1067
Neurl1a	1627	1469	1350	703	669	764
Nov	19354	24707	29190	1347	12196	3050
Fos	29190	50675	42848	10882	3786	3835

Table S2. *Erbb2* target genes are enriched in untreated P1 samples compared to P4.
IPA Analysis of upstream regulators revealed *Erbb2* target genes that were differentially expressed between P1 and P4 in the absence of treatment, implying enriched proliferative capacity at an early postnatal time-point. Samples are divided by significance of their calculated adjusted p-value; p-adjusted ≤ 0.05.

Upregulated at P1, p < 0.05; FC > 1.25				Upregulated at P4, p < 0.05; FC > 1.25			
	Fold Change		Fold Change		Fold Change		Fold Change
Aspm	8.77	*Vim*	1.81	*Adam8*	9.21	*Hey1*	1.36
Cxcl9	7.19	*Sp5*	1.81	*Bcl3*	8.08	*Spint1*	1.36
Has2	6.99	*S100a6*	1.81	*Ar*	7.24	*Dnajb6*	1.35
Fos	6.64	*Lamc2*	1.80	*Tns4*	6.71	*Ebp*	1.35
hp	5.69	*Sparcl1*	1.80	*Lgals3*	5.98	*Cd9*	1.34
Egr1	5.61	*Emp1*	1.78	*Foxa1*	5.73	*Sec61a1*	1.34
Clec3b	5.31	*Hspb8*	1.77	*Edn1*	5.25	*Gpd2*	1.33
Ccnb1	5.22	*Adamts16*	1.77	*G6pd2*	4.25	*Chd4*	1.32
Ccnb2	5.12	*Cks1b*	1.77	*Fgf7*	4.08	*Wfs1*	1.32
Mme	4.98	*Net1*	1.77	*Plet1*	4.02	*Chuk*	1.31
Ehf	4.41	*Pcna*	1.76	*Thbd*	3.96	*Uck1*	1.29
Tgfb1	4.25	*Gpc1*	1.74	*Igfbp6*	3.95	*Cul1*	1.27
Ctgf	4.23	*Cdca7l*	1.73	*Igfbp4*	3.94	*Sparc*	1.27
Cdc25c	4.22	*Itga6*	1.72	*Lgals1*	3.74	*Fasn*	1.25
Kcnn4	3.94	*Jag2*	1.72	*Cdkn1a*	3.58		
Tnc	3.84	*Ghr*	1.72	*Foxo1*	3.33		
Klf4	3.76	*Cdca4*	1.70	*Dhh*	2.91		
Il25	3.71	*Abracl*	1.70	*Sorbs1*	2.90		
Cdca7	3.47	*Atf4*	1.70	*Mxi1*	2.86		
Vcan	3.26	*Nucb2*	1.68	*Pole*	2.79		
Shmt1	3.25	*Mycn*	1.64	*Map1b*	2.62		
Col6a2	3.09	*Nek2*	1.64	*Gata3*	2.46		
Agr2	3.04	*Ralb*	1.64	*Homer2*	2.38		
Wisp2	3.04	*Mcl1*	1.60	*Ptges*	2.26		
Traf1	3.02	*Angptl2*	1.57	*Nedd9*	2.18		
Mki67	2.93	*Anxa2*	1.56	*Sox4*	2.03		

Development • Supplementary information

Itga5	2.85	*Tubb4a*	1.56	*Adipor2*	1.91
Junb	2.72	*Mfap2*	1.54	*Id2*	1.91
Birc5	2.66	*Mcm4*	1.54	*Admats5*	1.90
Cdc42ep3	2.63	*Smtn*	1.54	*Egfr*	1.84
Abcg2	2.60	*Lamb1*	1.52	*St3gal6*	1.83
Cxcl12	2.60	*Wnt5a*	1.51	*Pik3cd*	1.79
Btg2	2.59	*Cdh2*	1.48	*Adam17*	1.78
Ptgs1	2.54	*Orc6*	1.48	*Itgb3*	1.77
Rrad	2.52	*Fez2*	1.46	*Cdc42ep2*	1.74
Aldh1a7	2.51	*Igf2*	1.45	*Chchd10*	1.73
Cdh11	2.47	*Rrm1*	1.43	*Gbe1*	1.72
Elf5	2.45	*Gls*	1.43	*Prom1*	1.72
Crip1	2.44	*Hmgb2*	1.42	*Lum*	1.70
Srpx	2.42	*Rhob*	1.42	*Vwf*	1.70
Bcl2	2.41	*Marcks*	1.42	*Ndst1*	1.69
Zfp36l2	2.37	*Cdc25b*	1.41	*Lig1*	1.68
Skp2	2.36	*Rlp17*	1.41	*Vcl*	1.67
Thsd1	2.34	*Ehhadh*	1.41	*Tgfa*	1.63
Col6a3	2.32	*Litaf*	1.40	*Socs2*	1.63
Gja1	2.32	*Pold2*	1.39	*Irf6*	1.63
Fstl1	2.31	*Flot2*	1.38	*Phyh*	1.63
Mcm5	2.30	*Prps1*	1.38	*Cyb561*	1.59
Col5a1	2.28	*Serp1*	1.37	*Npnt*	1.59
Tap1	2.25	*Alad*	1.37	*Galnt3*	1.59
Myl9	2.19	*Pgm1*	1.36	*Cdkn1b*	1.58
Cdh3	2.18	*Ccdc80*	1.36	*Itpr2*	1.56
Pdlim4	2.13	*Dusp18*	1.36	*Dhrs7*	1.56
Mmp16	2.10	*Polr3d*	1.35	*Gpam*	1.55
Col5a2	2.08	*Pdcd4*	1.35	*St3gal4*	1.55
Hes1	2.08	*Polg2*	1.35	*Nptx2*	1.52
Col4a1	2.06	*Bnip2*	1.34	*Gspt2*	1.50
Cdon	2.05	*Aimp2*	1.34	*Tuba1a*	1.50

Penk	2.04	*Daam1*	1.34	*Arhgef5*	1.49
Cadm1	2.03	*Ppat*	1.33	*Elf3*	1.49
Sult1a1	2.03	*Cebpb*	1.33	*Bcat2*	1.48
Jun	2.03	*Clu*	1.32	*Mmp11*	1.47
Cdc20	2.02	*Mif*	1.31	*Mapk8ip3*	1.46
Gins2	1.99	*Maoa*	1.30	*Jup*	1.46
Alox15	1.98	*Rps10*	1.30	*F2r*	1.43
Etv1	1.97	*Pfn2*	1.29	*Jak1*	1.42
Polr1b	1.92	*Lrrfip1*	1.28	*Pdpk1*	1.42
Gas6	1.92	*Poli*	1.28	*Strbp*	1.41
Pdia4	1.90	*Cdc23*	1.26	*Polb*	1.40
Slc1a6	1.89	*Bmp7*	1.26	*Lsr*	1.40
Cdt1	1.87	*Ddx10*	1.25	*Cdh7*	1.39
P4ha2	1.83	*Fen1*	1.25	*Cradd*	1.37
